# Transcription Factors in the Regulation of Leydig Cell Gene Expression and Function

**DOI:** 10.3389/fendo.2022.881309

**Published:** 2022-04-07

**Authors:** Karine de Mattos, Robert S. Viger, Jacques J. Tremblay

**Affiliations:** ^1^ Reproduction, Mother and Child Health, Centre de recherche du centre hospitalier universitaire de Québec, Université Laval, Québec City, QC, Canada; ^2^ Centre de recherche en Reproduction, Développement et Santé Intergénérationnelle, Department of Obstetrics, Gynecology, and Reproduction, Faculty of Medicine, Université Laval, Québec City, QC, Canada

**Keywords:** transcription factors, gene expression, regulatory element, DNA binding motif, steroidogenesis, Leydig cells

## Abstract

Cell differentiation and acquisition of specialized functions are inherent steps in events that lead to normal tissue development and function. These processes require accurate temporal, tissue, and cell-specific activation or repression of gene transcription. This is achieved by complex interactions between transcription factors that form a unique combinatorial code in each specialized cell type and in response to different physiological signals. Transcription factors typically act by binding to short, nucleotide-specific DNA sequences located in the promoter region of target genes. In males, Leydig cells play a crucial role in sex differentiation, health, and reproductive function from embryonic life to adulthood. To better understand the molecular mechanisms regulating Leydig cell differentiation and function, several transcription factors important to Leydig cells have been identified, including some previously unknown to this specialized cell type. This mini review summarizes the current knowledge on transcription factors in fetal and adult Leydig cells, describing their roles and mechanisms of action.

## 1 Introduction

Localized in the testicular interstitium, Leydig cells are the principal source of testosterone and insulin-like 3 (INSL3), two hormones that regulate male reproductive development and function. In mammals, there are at least two distinct populations of Leydig cells, fetal Leydig cells (FLC) and adult Leydig cells (ALC), which are responsible for the synthesis of steroid hormones in the prenatal and postnatal testes, respectively [reviewed in (1, 2)]. Steroidogenesis is a multi-step process requiring various transporters and enzymes to convert cholesterol into a steroid hormone [reviewed in (3)]. The expression of the genes coding for these steroidogenic proteins is finely regulated to avoid steroid hormone insufficiency or excess across the lifespan.

Transcription factors (TFs) are fundamental to the regulation of gene expression. They are specialized proteins that recognize and bind to regulatory DNA sequences, modulating the rate of gene transcription [reviewed in (4)]. TFs typically recruit or interact with other TFs forming a unique molecular code that is key for specifying temporal- and tissue-specific gene expression as well as hormone responsiveness in hormone-sensitive target tissues. Moreover, TFs exhibit a dynamic behaviour that is characterized by their ability to interact with various partner proteins and to regulate different target genes according to many determinants such as cell type, development stage, and signal stimulus, among others.

In recent years, the development of novel and powerful methodological approaches in molecular genetics has led to the emergence of new information regarding the role of TFs in the regulation of Leydig cell differentiation and function, and by extension, in male fertility and reproductive health. In this mini review, we provide a brief overview of the roles and mechanisms of action of some of the most characterized TFs in Leydig cells. We have adopted the most recent classification of TFs, which is based both on amino acid sequence homology and the tertiary structure of their DNA-binding domains (5). Using this classification, TFs that have been identified in Leydig cells are presented in **Table 1**; **Table 2** lists the target genes for these TFs in Leydig cells.

**Table 1:** Classification of transcription factors identified in Leydig cells.

Superclass	Class	Family	Subfamily	Transcription factor
Basic Domains	Basic leucine zipper factors (bZIP)	Jun-related	Jun	cJUN
				JUNB
			NF-E2-like factors	NFE2L2 (NRF2)
		Fos-related	Fos	cFOS
				FRA-2 (FOSL2)
		CREB-related	CREB-like	CREB
				CREM
		C/EBP-related	C/EBP	C/EBPβ
	Basic helix-loop-helix factors (bHLH)	PAS domain	Arnt-like factors	ARNTL (BMAL1)
		bHLH-ZIP	SREBP factors	SREBP
			USF	USF1
				USF2
			n.a.	SPZ1
	Basic helix-span-helix factors (bHSH)	AP-2	n.a.	AP-2
Zinc-Coordinating DNA-Binding Domains	Nuclear receptors with C4 zinc fingers	Steroid Hormone Receptors (NR3)	GR-like receptors (NR3C)	NR3C1 (GR)
				NR3C2 (MR)
				NR3C3 (PR)
				NR3C4 (AR)
			ER-like (NR3A)	Erα; Erβ
		Thyroid hormone receptor-related (NR1)	Retinoic acid receptors (RAR - NR1B)	RARα, RARβ, RARγ
			Thyroid hormone receptors (THR - NR1A)	TRα, TRβ
			PPAR (NR1C)	PPARα, PPARβ/δ, PPARγ
			LXR (NR1H)	LXRα, FXR
		RXR-related receptors (NR2)	Retinoid X receptors (NR2B)	RXRα, RXRβ, RXRβ
			Testicular receptors (NR2C)	TR2 (NR2C1)
			COUP-like receptors (NR2F)	COUP-TFII (NR2F2)
		NGFI-B-related receptors (NR4A)	n.a.	NR4A1 (NUR77, NGFI-B)
				NR4A2 (NURR1)
		FTZ-F1-related receptors (NR5A)	n.a.	NR5A1 (SF-1, FTZ-F1)
				NR5A2 (LRH1)
		DAX-related receptors (NR0B)	n.a.	NR0B1 (DAX1)
				NR0B2 (SHP)
	Others C4 zinc finger-type factors	GATA-type zinc fingers	Two zinc-finger GATA factors	GATA4
	C2H2 zinc finger factors	Three-zinc finger Krüppel-related	Sp1-like	SP1
				SP3
			Kr-like	KLF6
			EGR	EGR1 (NGFI-A)
		More than 3 adjacent zinc fingers	ZNF44-2-like	ZNF44 (GIOT2)
			(unclassified)	ZNF461 (GIOT1)
Helix-Turn-Helix domains	Homeodomain factors	Paired-related HD	ARX	ARX
			RHOX	RHOX4
				PBX1
		HD-LIM	LHX2-like	LHX9
	Fork head/winged helix factors	Forkhead box (FOX)	FOXA	FOXA3 (HNF-3γ)
Alpha-helices exposed by beta-structures	MADS box factors	Regulators of differentiation	MEF2	MEF2A
				MEF2C
				MEF2D
Immunoglobulin fold	Rel homology region (RHR) factors	NF-kappaβ-related	NF-kappaβ p50 subunit-like	NF-κβ p50
			NF-kappaβ p65 subunit-like	NF-κβ p65 (RelA)
	STAT domain factors	STAT	n.a.	STAT5B

n.a., not applicable.

**Table 2:** Transcription factors and their target genes in Leydig cells.

Transcription Factor	Target Gene*	Select References
AP-1 (cJUN/cFOS)	*h, mStar*	(6–10)
	*mGja1*	(11)
	*mFdx1*	(12)
CREB/CREM	*mStar*	(10, 13–15)
	*hCKLFSF2B*	(16)
C/EBPβ	*mStar*	(17–19)
	*rNr4a1 (Nur77)*	(20)
BMAL1	*mStar*	(21)
AP-2	*m, rLhr*	(22, 23)
NR2F2 (COUP-TFII)	*mStar*	(24)
	*mInsl3*	(25)
	*mAmhr2*	(26)
	*mAkr1c14*	(27)
	*mGsta3*	(28)
	*mInha*	(28)
NR4A1 (NUR77, NGFI-B)	*mStar*	(9, 29, 30)
	*m, hHsd3b*	(31, 32)
	*h, mInsl3*	(33, 34)
	*rCyp17a1*	(35, 36)
NR5A1 (SF1, FTZ-F1)	*m, hStar*	(9, 15, 17–19, 37)
	*rCyp19a1*	(38)
	*hHSD3B2*	(32)
	*hCyp11a1*	(37)
	*rCyp17a1*	(39, 40)
	*rPrlr*	(41)
	*rAmhr2*	(42)
	*mVanin-1*	(43)
	*m, hInsl3*	(33, 34)
	*mFdx1*	(12)
NR5A2 (LRH1)	*mStar*	(9)
	*rCyp19a1*	(44)
	*m, hInsl3*	(33)
NR0B1 (DAX1)	*mStar*	(45)
GATA4	*h, mStar*	(7, 18, 19, 46, 47)
	*hHSD3B2*	(32)
	*mAmhr2*	(46)
	*rSrd5a1*	(46)
SP1	*rSrbi*	(48)
	*mLhr*	(22)
SP1/SP3	*mVegf*	(49)
	*mPbr*	(50)
KLF6	*hINSL3*	(34)
FOXA3 (HNF-3γ)	*rPdgfra*	(51)
MEF2	*mStar*	(47)
	*rNr4a1 (Nur77)*	(52)
	*mGsta1-4*	(53)
	*mAkr1c14*	(27)
NF-κβ p50	*rNr4a1 (Nur77)*	(20)
NF-κβ p65 (RelA)	*rCyp17a1*	(31)
STAT5B	*mStar*	(8)
	*rNr4a1 (Nur77)*	(8)

^*^The letter preceding the name of the gene refers to the species: m, mouse; r, rat; h, human.

## 2 Superclass of Basic Domains

### 2.1 Class of Basic Leucine Zipper Factors (BZIP)

#### 2.1.1 AP-1 Factors

The activator protein 1 (AP-1) is a dimeric complex that includes members of the JUN, FOS, activating transcription factor (ATF), and musculoaponeurotic fibrosarcoma (MAF) families of TFs (54). Among the AP-1 members, JUN and FOS are the best characterized. The JUN subfamily comprises three members (cJUN, JUNB, and JUND) while four members compose the FOS subfamily [cFOS, FOSB, Fos-related antigens 1 (FRA-1, FOSL1), and Fos-related antigens 2 (FRA-2, FOSL2)]. Members of the JUN family can homodimerize or heterodimerize, whereas FOS family members only form heterodimers. The DNA sequence recognized by AP-1 members differs according to the dimer involved. JUN : JUN and FOS : JUN dimers recognize the TPA-response element (TRE; TGA(C/G)TCA) and the cAMP-responsive element (CRE; TGACGTCA), whereas ATF dimers preferentially recognize the CRE motif, and MAF dimers bind to MAF recognition elements (MAREs), a long palindromic sequence that contains TRE or CRE motifs (55) [reviewed in (56)].

AP-1 members were first described in Leydig cells in the late 1990s (57). AP-1 factors regulate several genes in Leydig cells such as the steroidogenic acute regulatory protein (*Star*) gene, which is activated by cJUN (6, 7, 12). In addition, cJUN cooperates with other TFs, including GATA4, STAT5B, and NUR77 leading to a stronger activation of the *Star* promoter (7–9). Both cJUN and cFOS regulate *Star* promoter activity by recruiting CREB and CBP (10). Transcription of the gap junction protein alpha1 [*Gja1*, also known as connexin43 (*Cx43*)] gene, involved in the initiation and maintenance of sperm production, is also controlled by cJUN, JUNB, and FOSL2, and by a cJUN/cFOS cooperation (11, 58). Furthermore, the ferredoxin 1 (*Fdx1*) promoter is activated by a cJUN/SF1 cooperation (12). *Fdx1* is a partner of *Cyp11a1*, participating in the conversion of cholesterol into pregnenolone, the first and rate-limiting step in steroidogenesis. It is important to note that the nature of the cJUN dimerization partner influences its role in gene regulation. For example, the combination of either FOSL2 or cFOS with cJUN inhibits the stimulatory effect of cJUN on the *Star* promoter (6, 10, 59). AP-1 factors in Leydig cells have been reviewed elsewhere (56).

#### 2.1.2 CREB-Related Factors

CREB-related factors include three members: CRE-binding protein (CREB), cAMP response element modulator (CREM), and CRE-activating transcription factor (ATF-1). CREB factors homodimerize and heterodimerize with other CREB members and with other bZIP TFs, such as AP-1 members (60). CREB factors regulate transcription by binding to a CRE motif (TGACGTCA) similar to that recognized by AP-1 members, leading to overlap and redundancy in their activities (61). Although CREM is the most abundant member in MA-10 Leydig cells, all CREB members activate *Star* transcription through CRE elements located in the proximal promoter region (13, 14). Moreover, CREB factors cooperate with SF1 (NR5A1, Ad4BP) to enhance *Star* transcription (15). CREB also stimulates *CKLFSF2B* promoter activity in response to LH/cAMP (16). *Cklfsf2b* codes for a protein that inhibits steroidogenesis in Leydig cells (16). Therefore, CREB is involved in both activation and repression of steroidogenesis in Leydig cells depending on its target genes.

#### 2.1.3 C/EBP-Related Factors

Members of the CCAAT/enhancer binding protein (C/EBP) subfamily contain a bZIP DNA-binding domain and regulate gene expression by binding to the sequence (A/G)TTGCG(C/T)AA(C/T) as homo- or heterodimers (62). C/EBPβ is the predominant member in Leydig cells (17, 63) where it activates *Star* transcription alone and in cooperation with SF1 and GATA4 (17–19). C/EBPβ also cooperates with NF-κβ p50 to stimulate *Nur77* promoter activity in Leydig cells (20). The *Nur77* gene encodes the orphan nuclear receptor NUR77, which regulates several genes involved in steroidogenesis in Leydig cells (see Section 3.1.2, *NGFI-B/NR4A Receptors*, below).

## 3 Superclass of Zinc-Coordinating DNA-Binding Domains

### 3.1 Class of Nuclear Receptors With C4 Zinc Fingers

TFs belonging to the nuclear receptor class respond to extracellular and intracellular signals to regulate gene expression. They also regulate cellular functions within the cytoplasm (64). In this section we present the nuclear receptors for which the roles and mechanisms of action are, or have begun to be, characterized in Leydig cells. Detailed information can be found in a review article dedicated to nuclear receptors in Leydig cells (65).

#### 3.1.1 COUP-Like/NR2F Receptors

The nuclear receptor subclass 2, group F (NR2F) subfamily consists of three members: chicken ovalbumin upstream promoter transcription factor I (COUP-TFI, NR2F1, EAR3), COUP-TFII (NR2F2, ARP1) and COUP-TFIII (NR2F6, EAR2). NR2Fs have been implicated in various physiological and developmental processes by regulating the expression of numerous genes [reviewed in (66, 67)]. *Via* their double zinc finger DNA-binding domain, NR2F factors bind as monomers to the nuclear receptor element AGGTCA and its variants. They also bind as dimers to direct (DR), inverted (IR), and everted (ER) repeats separated by 1-12 nucleotides (68).

Of the NR2F subfamily members, COUP-TFII is by far the most abundant in Leydig cells. Although COUP-TFII is present in mice interstitial cells from early fetal life throughout adulthood, it is only associated with steroidogenically active ALC in postnatal life (24). COUP-TFII is a marker of stem cells giving rise to the ALC population (24, 69). *In vivo* studies using mouse models have shown that COUP-TFII is crucial for Leydig cell development and male reproductive function (70, 71). In Leydig cells, COUP-TFII regulates the expression of several genes involved in lipid metabolism, male gonad development, and steroidogenesis (28). COUP-TFII activates *Star*, *Insl3*, and *Amhr2* expression by binding to their respective promoter sequences (24–26). It cooperates with SF1 on the *Star* and *Insl3* promoters (24, 25) and with SP1 on the *Amhr2* promoter (26). The *Akr1c14* gene, which codes for the 3α-HSD enzyme that catalyzes the interconversion of dihydrotestosterone (DHT) into 5α-androstane-3α,17β-diol (3α-diol), is activated by COUP-TFII in cooperation with MEF2 (27). COUP-TFII also activates the expression of *Gsta3* and *Inha*, genes involved in the inactivation of reactive oxygen species and in the homeostasis of the hypothalamic-pituitary-gonadal axis, respectively (28). Expression of several other Leydig cell genes including *Cyp17a1*, *Hsd3b1* and *Cyp11a1* is reduced in *Coup-tfii* null mice (71) and in COUP-TFII-depleted MA-10 Leydig cells (28), implying a role for COUP-TFII in their expression.

#### 3.1.2 NGFI-B/NR4A Receptors

The NR4A family consists of three orphan nuclear receptors: neuron-derived clone 77 (NR4A1, NUR77, NGFI-B, TR3), nuclear receptor related 1 (NR4A2, NURR1) and neuron-derived orphan receptor 1 (NR4A3, NOR1). NR4A members can bind to DNA either as monomers, homodimers, or heterodimers. NUR77 and NURR1 also heterodimerize with RXR. As monomers, they bind to a NGFI-B-response element (NBRE; AAAGGTCA), as homodimers and heterodimers to a Nur-response element (NurRE; TGATATTTN_6_AAATGCCA), and as heterodimers with RXR to a DR5 sequence [reviewed in (72, 73)]. NR4A factors are immediate early response genes involved in the regulation of several physiological and pathological processes, including steroidogenesis (74) [reviewed in (75)].

Leydig cells contain mainly NUR77, followed by NURR1 where both are important regulators of basal and hormone-induced gene transcription (76). *Nur77* expression is strongly increased by LH (76) *via* the CAMKI pathway (29, 77) consistent with its role as a key regulator of several genes in Leydig cells including *Cyp17a1* (31, 35), *Hsd3b* (31), *HSD3B2* (32), *Insl3* (33, 34), and *Star* (29, 30). NUR77 regulates the expression of these genes by cooperating with CAMKI (29), cJUN (9), KLF6 (34), and SF1 (34). In Leydig cells, *Nur77* expression is controlled by distinct regulatory elements for both basal and hormone-induced expression (77), through mechanisms involving MEF2 (52), STAT5B (8), CREB (77), cJUN (9), C/EBPβ (20), and NF-κβ p50 (20).

#### 3.1.3 FTZ-F1-Related/NR5A Receptors

The nuclear receptor 5A (NR5A) family comprises two members: steroidogenic factor 1 (NR5A1, Ad4BP, SF1) and liver receptor homolog 1 (NR5A2, LRH1, FTF). Both factors share high sequence similarity, bind to the same DNA motif, regulate common target steroidogenic genes, and exhibit overlapping expression in several tissues [reviewed in (78, 79)]. Despite this, they have nonredundant roles and cannot fully compensate for each other [reviewed in (78, 79)]. NR5A members regulate gene expression by binding as monomers to the sequence (T/C)CAAGGTCA located in the promoter region of target genes.

SF1 was initially identified as a tissue-specific activator of several cytochrome P450 steroid hydroxylase genes (38, 80). SF1 is essential for steroidogenesis, reproduction, and male sex differentiation, as revealed by mutations in the *SF1* gene in humans and in mouse models where adrenal and gonadal development and function are impaired (37, 81–84) [reviewed in (85, 86)]. Interestingly, *Sf1* knockdown in MLTC-1 Leydig cells leads to downregulation of *Star* and *Cyp11a1* and accumulation of neutral lipids and cholesterol (37). Moreover, SF1 is one of only a handful of TFs that can convert fibroblasts into functional Leydig-like cells, revealing the pivotal role of this nuclear receptor in Leydig cells (87, 88).

*In vitro* analysis of regulatory elements has shown that the expression of several Leydig cell genes is regulated by SF1. These include *Star* (9, 17, 37), *Cyp19a1* (38), *HSD3B2* (32), *Cyp17a1* (39, 40), *Cyp11a1* (37), *Prlr* (41), *Amhr2* (42), *Vanin-1* (43), *Insl3* (33), and *Fdx1* (12). SF1 activity relies on interactions with a long list of protein partners, such as C/EBPβ (17), cJUN (9, 12), DAX1 (45), GATA4 (89), and KLF6 (34).

Like SF1, LRH1 influences steroidogenesis and fertility. To date, only a few genes are known to be regulated by LRH1 in Leydig cells, including *Star* (in cooperation with cJUN) (9), *Cyp19a1* (44), and *Insl3* (33).

#### 3.1.4 DAX-Related/NR0B Receptors

The DAX-related receptor (NR0B) family comprises two members: critical region on the X chromosome gene 1 (NR0B1, DAX1) and small heterodimer partner (NR0B2, SHP). They lack the typical zinc finger DNA-binding domain and therefore act mainly as transcriptional repressors by inhibiting the activity of other TFs (90, 91). Both members are present in Leydig cells and act as homodimers or heterodimers (92).

In *Dax1*-deficient mice, testis cord organization is compromised and FLC development is arrested (93). *In vitro* studies in Leydig cell lines revealed that DAX1 represses steroidogenesis by inhibiting *Star* expression, while silencing *Dax1* expression increases *Star* transcription leading to enhanced steroidogenesis (45). DAX1 interacts with and represses the activity of NUR77 and SF1, inhibiting *Star* expression (36, 45). Interestingly, *Dax1* knockdown in MA-10 Leydig cells decreases *Cyp11a1* and *Star* expression suggesting that DAX1 could also act as a coactivator in addition to its repressor role (94).

SHP is a repressor of steroidogenesis. In mouse Leydig cells, *Shp* expression is reduced by hCG treatment (95). In *Shp-*deficient mice, testosterone levels as well as *Star*, *Cyp11a1*, and *Hsd3b1* mRNA levels are increased leading to premature sexual maturation (96). SHP inhibits steroidogenesis by interacting and repressing the activity of LHR1 (96). *Shp* mRNA levels are significantly reduced in COUP-TFII- and MEF2-depleted Leydig cells, indicating that *Shp* expression requires these two TFs (28, 97).

### 3.2 Class of Other C4 Zinc Finger-Type Factors

#### 3.2.1 Two Zinc-Finger GATA Factors

The six GATA members (GATA1 to 6) are crucial for the development and function of several tissues, including the male gonad [reviewed in (98, 99)]. GATA factors regulate gene expression by binding *via* their two zinc fingers to the DNA sequence (A/T)GATA(A/G) in the promoter region of target genes. Of the six GATA factors, GATA4 is the most abundant in Leydig cells *in vivo* (100–102). Its expression is also the broadest being present from the onset of testis morphogenesis and into adult life (103). Considered one of the first gonadal markers in both sexes, GATA4 is required for urogenital ridge development in mice and later for mammalian gonadal differentiation (103, 104).

A *Sf1*-Cre mouse line, which expresses the Cre recombinase in several tissues including Leydig, Sertoli and adrenal cells, was used to conditionally inactivate *Gata4*. The resulting males were undervirilized and had small testes lacking mature sperm (105), thereby supporting a role for this factor in male reproductive function. Transcriptomic analysis of GATA4-depleted MA-10 Leydig cells revealed several deregulated pathways, including cholesterol metabolism and steroidogenesis (46). Consistent with this, GATA4 stimulates the transcription of several genes expressed in Leydig cells such as *HSD3B2* (32), *Cyp19a1* (106), *Star* (46, 106), *Inha* (106), *Sf1* (106), *Amhr2* (46), and *Srd5a1* (46). GATA4 also cooperates with cJUN, C/EBPβ, and MEF2 to upregulate *Star* expression (7, 18, 47). These results emphasize the indispensable role of GATA4 in the differentiation and function of FLC and ALC (46, 107). The critical nature of GATA4 in the Leydig cell differentiation is further supported by the demonstration that GATA4, along with SF1 and DMRT1 or NUR77, are sufficient to reprogram fibroblasts toward the Leydig-like cell fate (87, 88).

## 4 Superclass of Helix-Turn-Helix Domains

### 4.1 Class of Forkhead/Winged Helix Factors

#### 4.1.1 Forkhead Box (FOX) Factors

The forkhead box A3 (FOXA3) is the only member of the FOXA subfamily present in the testes, mainly in ALC (51, 108, 109). So far, the only direct target identified for FOXA3 in Leydig cells is the gene coding for the platelet-derived growth factor receptor alpha (*Pdgfra*) (51), that in response to PDGF signaling, acts in Leydig cell differentiation and testis organogenesis (110). In cAMP-induced steroidogenesis, FOXA3 is proposed to repress *Nur77* expression, which in turn reduces steroidogenic gene expression and testosterone production (111). These findings indicate that FOXA3 participates actively in the control of Leydig cell function and male fertility.

## 5 Superclass of α-Helices Exposed by β-Structures

### 5.1 Class of MADS Box Factors

#### 5.1.1 MEF2 Subfamily

The Myocyte Enhancer Factor 2 (MEF2) factor subfamily comprises four members (MEF2A-2D) that share two highly conserved domains, a MADS box and a MEF2 domain, involved in dimerization and DNA binding [reviewed in (112)]. MEF2 factors form homo- and heterodimers that bind the sequence YTAWWWWTAR (Y=C/T, W=A/T, R=G/A) in the promoter region of their target genes. Because of their conserved DNA-binding domain, MEF2 members share common targets and can compensate for each other. MEF2 members also display unique spatiotemporal patterns in different tissues. Due to their divergent transactivation domain, MEF2 factors respond to different signals and interact with different partners, leading to specific gene expression [reviewed in (112)].

Although widely studied in other organs, the presence of MEF2 in the testes, more specifically in Sertoli and Leydig cells, was only reported in 2014 (52). In Leydig cells, MEF2A and MEF2D and to a lesser extent MEF2C, are expressed from early gonadal development into adulthood (52). MEF2A/2D-depleted MA-10 Leydig cells produce less steroid hormone demonstrating that MEF2 factors have a role in male reproductive function (47). Consistent with this, microarray analysis of MEF2A/2D-depleted MA-10 Leydig cells identified several differently regulated genes known to be involved in fertility, gonad morphology, and steroidogenesis (97). To date, direct gene targets for MEF2 factors in Leydig cells include *Nur77* (52), *Gsta1-4* (53), *Star* (involving a MEF2/GATA4 cooperation) (47), and *Akr1c14* (through a cooperation with COUP-TFII) (27). The complete network of genes regulated by MEF2 factors in Leydig cells as well as MEF2 interacting partners remain to be fully elucidated.

## 6 Superclass of Immunoglobulin Fold

### 6.1 Class of STAT Domain Factors

#### 6.1.1 STAT Factors

The signal transducer and activator of transcription (STAT) family consists of seven proteins [reviewed in (113)]. Cytokines and growth factors activate STAT members through the Janus kinase (JAK) signaling pathway. In the nucleus, STAT factors regulate gene transcription by binding as homo- or heterodimers to the γ-interferon-activated sequence (GAS; TTCN_3_GAA) in the promoter region of target genes. So far, STAT5B is the only STAT factor identified in Leydig cells (114). In these cells, STAT5B is activated by growth hormone, an important regulator of steroidogenesis (8). STAT5B activates *Star* expression directly by binding to a GAS element and in cooperation with cJUN (8). STAT5B also activates the *Nur77* promoter (8).

## 7 Other Transcription Factors Present in Leydig Cells

Other TFs have been described in Leydig cells, but their mechanisms of action remain poorly characterized. This includes the nuclear factor E2-related factor-2 (NRF2, NFE2l2), which is an important modulator of reactive oxygen species levels, especially in aging Leydig cells (115–117). Furthermore, the brain and muscle arnt-like protein-1 (BMAL1), a component of the circadian clock system, is also directly involved in the control of Leydig cell function in different species, by regulating the expression of *Star*, *Hsb3b*, and *Cyp11a1* (21, 118, 119). Finally, members of the nuclear factor kappa-beta (NF-κβ) family, involved in immune and inflammatory responses, also contribute to the regulation of steroidogenesis in Leydig cells (20, 31, 120).

## 8 Concluding Remarks

As described in this mini review, several TFs belonging to different classes and families are pivotal to ensure proper Leydig cell differentiation and function. This underscores the complex regulatory mechanisms involved. Most of the knowledge acquired so far has relied on *in vitro* analyses of regulatory elements of genes expressed in Leydig cells. Although we are far from fully understanding all the signals, pathways, and TFs involved, technological advances and novel mouse models will certainly lead to significant discoveries in the coming years.

## Author Contributions

KM wrote the first draft of the manuscript. All authors contributed to the article and approved the submitted version.

## Funding

Supported by a grant from the Canadian Institutes of Health Research (funding reference number MOP-81387) to JT. KM is the recipient of a studentship from the Fonds de recherche du Québec-Santé.

## Conflict of Interest

The authors declare that the research was conducted in the absence of any commercial or financial relationships that could be construed as a potential conflict of interest.

## Publisher’s Note

All claims expressed in this article are solely those of the authors and do not necessarily represent those of their affiliated organizations, or those of the publisher, the editors and the reviewers. Any product that may be evaluated in this article, or claim that may be made by its manufacturer, is not guaranteed or endorsed by the publisher.
